# Increasing participation in habitual intellectual activities on modulating functional connectivity of default mode network among older adults at risk of dementia: study protocol of a randomized controlled trial

**DOI:** 10.1186/s13063-022-06271-3

**Published:** 2022-04-14

**Authors:** Rachel W. K. Yan, Charlotte P. C. Kwok, Jessie O. T. Kwok, Kaspar K. W. Lee, Hanna Lu, Winnie C. W. Chu, Timothy C. Y. Kwok, Linda C. W. Lam, Allen T. C. Lee

**Affiliations:** 1 Department of Psychiatry, Faculty of Medicine, The Chinese University of Hong Kong, Hong Kong, SAR China; 2 Department of Imaging and Interventional Radiology, Faculty of Medicine, The Chinese University of Hong Kong, Hong Kong, SAR China; 3 Department of Medicine and Therapeutics, Faculty of Medicine, The Chinese University of Hong Kong, Hong Kong, SAR China

**Keywords:** Intellectual activities, Cognitive training, Calligraphy, Functional connectivity, Default mode network, Brain network, Subjective cognitive decline, Cognitive impairment, Cognitive maintenance, Dementia prevention

## Abstract

**Background:**

Active participation in intellectual leisure activities such as calligraphy helps prevent cognitive decline and dementia, but the underlying mechanisms are not fully understood. With disrupted functional connectivity (FC) of default mode network (DMN) associated with cognitive decline, we speculate that intellectual activities might optimize cognitive function through modulating FC of DMN. This two-arm single-blind randomized controlled trial aims to identify the effects of increasing practice of calligraphy on cognitive function and FC of DMN in people with subjective cognitive decline (SCD).

**Methods:**

One hundred twelve community-living Chinese aged 55 to 75 years old with SCD but without mild cognitive impairment or dementia and with prior practice of calligraphy as defined by 1 h of calligraphy per week will be recruited through elderly social centres in Hong Kong and randomized into either control or intervention group. The control group will continue with their usual practice of calligraphy, whereas the intervention group will double their practice of calligraphy. Measurement of cognitive outcomes and neuroimaging on resting-state FC will be performed at baseline and in 6 months. Repeated measures analysis of variance will be used to assess cognitive and FC changes, with time being the within-group factor, control/intervention as the between-group measure, and important covariates (age, sex, educational and occupational attainment, health, and other lifestyle factors) controlled for.

**Discussion:**

This study will shed light on the underlying neurocognitive mechanisms of how intellectual activities promotes cognitive maintenance. Our anticipated findings will provide evidence that reversing or slowing FC disruption by actively participating in intellectual activities is still possible for the at-risk individuals.

**Trial registration:**

Chinese Clinical Trial Registry ChiCTR1900024433. Registered on 11 July 2019.

## Administrative information

Note: the numbers in curly brackets in this protocol refer to SPIRIT checklist item numbers. The order of the items has been modified to group similar items (see http://www.equator-network.org/reporting-guidelines/spirit-2013-statement-defining-standard-protocol-items-for-clinical-trials/).

**Table:** 

Title {1}	Increasing participation in habitual intellectual activities on modulating functional connectivity of default mode network among older adults at risk of dementia: study protocol of a randomized controlled trial
Trial registration {2a and 2b}.	Chinese Clinical Trial Registry (ChiCTR1900024433), registered on 11.07.2019.
Protocol version {3}	Original version dated 30.12.2018 First revision dated 28.02.2019 • Primary reason for revision: Adding supplementary photos to respond to ethics committee’s query of whether calligraphy is commonly practiced in community-living older adults.
Funding {4}	Fully funded by Research Grants Council, University Grants Committee, Hong Kong. The funder has no role in the design of the study and collection, analysis, interpretation of data, and in writing the manuscript.
Author details {5a}	Rachel W. K. Yan,^1^ Charlotte P. C. Kwok,^1^ Jessie O. T. Kwok,^1^ Kaspar K. W. Lee,^1^ Hanna Lu,^1^ Winnie C. W. Chu,^2^ Timothy C. Y. Kwok,^3^ Linda C. W. Lam,^1^ Allen T. C. Lee^1^ ^1^Department of Psychiatry, Faculty of Medicine, The Chinese University of Hong Kong, Hong Kong SAR, China ^2^Department of Imaging and Interventional Radiology, Faculty of Medicine, The Chinese University of Hong Kong, Hong Kong SAR, China ^3^Department of Medicine and Therapeutics, Faculty of Medicine, The Chinese University of Hong Kong, Hong Kong SAR, China
Name and contact information for the trial sponsor {5b}	Nil
Role of sponsor {5c}	Nil

## Introduction

### Background and rationale {6a}

Dementia is a major health concern worldwide [1]. Given the rapidly ageing populations and the current absence of cost-effective disease-modifying treatment, finding ways to help at-risk individuals achieve cognitive maintenance and healthy ageing is of great clinical and public health importance [2–4].

Lifestyle modification such as participation in intellectual activities is increasingly recognized to be beneficial to cognition [5–14]. Our previous 6-year follow-up study of a large cohort of community-living dementia-free older adults found that while many reported engaging in leisure activities in their daily lives, those participating more in intellectual activities such as calligraphy, painting, reading books, and playing board games, cards and Mahjong were at a lower risk of incident dementia, independent of educational attainment, other health behaviours such as social and physical activities, physical and psychiatric comorbidities, and sensory impairments [15]. Higher level of participation in intellectual activities might serve as a useful, economical, and relatively safe non-pharmacological intervention for achieving cognitive maintenance in the at-risk older adults. Nevertheless, the underlying mechanisms of how intellectual activities slow cognitive decline are not fully understood [16].

Default mode network (DMN) is an interconnected set of different brain regions including precuneus/posterior cingulate cortex (PCC), medial prefrontal cortex (mPFC), lateral temporal cortex (LTC), inferior parietal lobe (IPL), and hippocampal formation which includes a proportion of parahippocampal cortex (HF+). It is most active when a person is at resting state (such as daydreaming and mind-wandering) but is deactivated when a person is engaged in external goal-orientated tasks [17]. Apart from serving as a sentinel which maintains a broad low-level focus of attention that monitors the external world for unexpected events, it is hypothesized to play a major role in thinking of self (e.g. self-reflection and autobiographical memory), mentalization (theory of mind, moral reasoning, and judgment), and remembering the past and envisioning the future [18].

With the help of resting-state functional magnetic resonance imaging (rs-fMRI), it is now recognized that DMN is organized into hubs and subsystems, with the activity of each brain region being highly correlated with the other when involving in the above functions. In particular, PCC serves as a hub that is activated in nearly all of the tasks described above; mPFC is involved in self-processing, mentalization, envisioning future goals, and decision-making (i.e. executive function, social cognition and flexibility in terms of higher cognitive function); LTC and HF+ are involved in memory; and IPL is involved in attention [18].

More importantly, different studies have shown that disruption of DMN, such as decreased functional connectivity (FC) in PCC and HF+, is present in people with clinical Alzheimer’s disease (AD) [19, 20]. It matches with not only the clinical presentation (i.e. poor memory and attention) but also other pathophysiological features of AD (e.g. hippocampal and medial temporal lobe atrophy as shown in structural MRI, and hypometabolism and beta-amyloid plaque accumulation in regions that overlap with disrupted DMN as shown in positron emission tomography) [20, 21]. Recently, increasing evidence shows that disruption of DMN is detected even in subjective cognitive decline (SCD), with decreased FC beginning in the posterior DMN, and becomes more pronounced and gradually progresses to other regions in mild cognitive impairment (MCI) [22–24]. These suggest that FC disruption appears very early in the course of AD and might be used in conjunction with other biomarkers to identify the severity and predict the progression of AD.

Given the importance of FC changes in the development of dementia, we propose to conduct a randomized controlled trial (RCT), with the aim to test whether increasing participation in habitual intellectual activities promotes cognitive maintenance via strengthening FC of DMN. In this study, calligraphy is chosen as the activity of interest, given its high prevalence of practice among cognitively active older adults in our locality and its popularity among those attending the elderly social centres. It has simultaneous involvement with different cognitive domains including attention and concentration, memory, spatial orientation, executive function, and eye-hand coordination, with evidence of improving global cognitive function [25, 26]. We will compare changes in cognitive function and FC following doubling of the usual practice of calligraphy for 6 months in community-living Chinese adults with SCD and prior practice of calligraphy.

Our study will shed light on the underlying neurocognitive mechanisms of how intellectual activities modulates cognitive function. More importantly, our anticipated findings may provide evidence that reversing, slowing, and/or compensating for FC disruption is still possible in SCD despite network dysfunction may have already been present in this early stage of AD. These will help support and extend previous literature that having greater participation in intellectual activities could potentially optimize cognitive reserve and strengthen resilience towards cognitive decline.

### Objectives {7}

The objectives of this study are:

1. To examine the effect of doubling the usual practice of calligraphy on global cognitive function in people with SCD;
2. To examine the effect of doubling the usual practice of calligraphy on FC within DMN in people with SCD; and
3. To examine the correlation between cognitive and FC changes.

### Trial design {8}

This study is a two-arm, parallel-design, single-blind RCT of 6-month calligraphy practice conducted in Hong Kong. Participants will be randomized in a 1:1 ratio to either the intervention or control group. The framework of our trial is to test the superiority of doubling the “dosage” of calligraphy practice (*vs* maintaining on the same “dosage”) on cognitive function and FC of DMN.

## Methods: Participants, interventions and outcomes

### Study setting {9}

Community-living Chinese individuals aged 55 to 75 years old with SCD and prior practice of calligraphy will be recruited through advertisement in elderly social centres in Hong Kong. Participants will practise calligraphy in their usual setting, either at their own home or in elderly social centres.

### Eligibility criteria {10}

#### Inclusion criteria

Before recruitment, potential participants will be screened for presence of SCD as defined by (i) Hong Kong Chinese version of Montreal Cognitive Assessment (HK-MoCA) score of > 25, which is above the cutoff scores for MCI in younger-old adults with high education level [27], and (ii) Abbreviated Memory Inventory for Chinese (AMIC) with at least 1 positive answer to the 5 screening questions [28].

In addition, the potential participants will be screened for any practice of calligraphy, as defined by having at least 1 h of calligraphy per week, in the previous 6 months. There is no restriction on the form of calligraphy, but they are expected to have their calligraphy practice in a quiet environment, and their calligraphic writing should involve brush handwriting by tracing the strokes and structures of the characters displayed in a mixture of traditional calligraphic styles [29].

#### Exclusion criteria

Exclusion criteria include non-Chinese ethnicity; living in care homes; having history of stroke, traumatic brain injury, Parkinson’s disease, MCI, or clinical dementia; taking drugs that affect cognition (e.g. benzodiazepines, anti-dementia medication, etc.); having history of depression, bipolar affective disorder, psychosis, or substance abuse; already attending other cognitive intervention programs or having regular participation in painting (at least 1 h of painting per week); significant impairments in communication or writing; and having no prior practice of calligraphy.

### Who will take informed consent? {26a}

Trained research assistant will obtain written informed consent from all participants before participation. Our research assistant will first explain to eligible participants our programme in detail (aims, requirements, length of study, etc.). Information sheet will be provided so that eligible participants have a clear idea of the expected commitment before deciding to participate. Written informed consent will be obtained prior to participation.

### Additional consent provisions for collection and use of participant data and biological specimens {26b}

All participants will be screened for any potential contraindications to plain MRI brain scan (metallic implant, pacemaker, and claustrophobia) and explained about the MRI brain scan as part of our baseline and follow-up assessment. Those who have contraindications or refuse to receive MRI brain scan will not be recruited. Information sheet will be provided so that eligible participants have a clear understanding of the need to undergo plain MRI brain scan. Written informed consent for MRI brain scan will be obtained prior to participation. This trial does not involve collecting biological specimens for storage.

## Interventions

### Explanation for the choice of comparators {6b}

The aim of this study is to test if increasing habitual participation in intellectual activities will optimize cognitive function and brain network in older adults. Therefore, we design the intervention as doubling the usual practice of calligraphy and the control as maintaining the usual practice.

### Intervention description {11a}

Participants will be randomized in a 1:1 ratio to one of the following arms.

- Control group: Participants will continue their usual practice of calligraphy in the same manner (i.e. same frequency, duration, style, and environment).
- Intervention group: Participants will continue their usual practice of calligraphy in the same manner, with the exception of doubling the amount of time spent in calligraphy.

### Criteria for discontinuing or modifying allocated interventions {11b}

Adverse events are expected to be minimal in this study, as no drug treatment or invasive procedure is involved, and the intervention (calligraphy) is already familiarized to the participants. Nevertheless, mild discomforts such as fatigue or muscle soreness due to prolonged sitting and writing and headache or eye soreness due intense concentration in calligraphy practice may be experienced by some participants. These potential discomforts will be explained to participants before signing consent. Participants will be advised to report to our research assistant any health problems or suspected adverse events during the study period. All complaints with regard to our intervention will be documented, and any adverse events potentially due to our intervention will be assessed and followed by the principal investigator. The intervention will be stopped immediately if the reported event is considered to be related.

### Strategies to improve adherence to interventions {11c}

To ensure compliance of calligraphy practice during the 6-month intervention period, participants from both groups will be asked to WhatsApp or email the research assistant a snapshot of their work and the amount of time they spend in calligraphy each time they finish practicing. Upon receiving their work, the research assistant will acknowledge receipt of their work and log but will not give them any feedback on their work. Those who do not have smartphones or internet access will be asked to record in a diary the date and amount of time spent on their work each time they finish practising calligraphy, and to hand in their diary and work to the research assistant bi-monthly for review of compliance.

### Relevant concomitant care permitted or prohibited during the trial {11d}

Individuals already attending other cognitive intervention programs will be excluded from participating in this study. Participants will be advised to continue their usual lifestyle, with the control group also maintaining on their habitual practice of calligraphy but the intervention group doubling on their calligraphy practice. They will also be advised not to attend other intervention programs or take medications that might affect their cognition and confound the results (e.g. brain tonics) during the study period.

### Provisions for post-trial care {30}

Serious adverse events or permanent harm done to the participants are highly unlikely in this study, as no drug treatment or invasive procedure is involved, and the participants have already been practising the intervention (calligraphy) prior to our study. Mild discomforts as described earlier are likely to be self-limiting and disappear with rest. Nevertheless, all complaints with regard to our intervention will be documented, and any adverse events potentially due to our intervention will be assessed and followed by the principal investigator. The intervention will be stopped immediately if the reported event is considered to be related, with referral to seek medical attention provided by the principal investigator.

### Outcomes {12}

#### Primary cognitive outcome

Global cognition will be assessed by the Chinese version of the Alzheimer’s Disease Assessment Scale-Cognitive Subscale (ADAS-Cog) [30], an objective cognitive test widely used in assessing global cognition even at the milder stages of AD progression.

#### Secondary cognitive outcomes

To increase the sensitivity of detecting important cognitive changes in our participants, additional assessments will be performed to assess the global and specific cognitive domains. Global cognition will be assessed by the HK-MoCA, a locally validated cognitive screening test to detect early memory and executive deficits in MCI [27], and the Memory Inventory for Chinese (MIC), which is a locally validated 27-item questionnaire on detecting SCD [31]. Regarding the specific cognitive domains, attention will be assessed by the digit span forward (DSF) test and the part A of trail making test (TMT-A); learning and memory will be assessed by the 10-min delayed recall and digit span backward (DSB) tests; and executive functioning will be assessed by the part B of trial making test and the Chinese verbal fluency test (CVFT) [32–36].

#### Primary neuroimaging outcome

Assessment and analysis of FC of DMN will be conducted based on the protocols developed by our team as reported previously [37, 38].

##### Data acquisition

1. Structural MRI (sMRI): Participants will be scanned using a 3 T MRI scanner with an 8-channel SENSE head coil (Achieva, Philips Medical Systems). A series of 3D high-resolution T1-weighted structural images will be collected for each participant with the following parameters: repetition time (TR) 7.4 ms, echo time (TE) 3.4 ms, flip angle 8°, voxel size 1.04× 1.04× 0.6 mm^3^, total scan time 8 min.
2. Resting-state functional MRI (rs-fMRI): During the rs-fMRI session, participants will be instructed to relax, stay awake, and hold still without falling asleep. In each scanning sequence, a series of 40 axial T2- weighted gradient echo-planar images (TR= 3000 ms, TE=25 ms, flip angle=90°, field of view (FOV)=230× 230 mm^2^, in-plane resolution=2.4× 2.4 mm^2^, matrix=96× 96, slice thickness=3 mm) will be captured. A total of 120 time points of rs-fMRI will be acquired for each participant.

##### Data preprocessing and analysis

Preprocessing of rs-fMRI images will be conducted by Statistical Parametric Mapping software (SPM12, http://www.fil.ion.ucl.ac.uk/spm/software/spm12/) and resting-state fMRI data analysis toolkit (REST, http://restfmri.net/forum/REST_V1.8) [39] embedded in MATLAB R2016a. The first 5 volumes of each functional time series will be discarded because of the instability of the initial MRI signal and initial adaption of participants to the situation. The remaining rs-MRI data will be subsequently corrected for slice timing and realigned to the first image by rigid-body head movement correction. Then, the rs-fMRI images are coregistered to high-resolution T1 images and normalized to standard stereotaxic anatomical Montreal Neurological Institute (MNI) space. The normalized volumes are spatially smoothed using an isotropic Gaussian filter of 8-mm full width at half maximum. The time course in each voxel will be detrended to correct for linear drift over time. Nuisance signal (whole brain, white matter, cerebrospinal fluid and six motion parameters) will be regressed out from the rs-fMRI time series. Temporal filtering with a band of 0.01–0.08 Hz will then be conducted to the time series of each voxel to reduce the impact of low-frequency drifts and high-frequency noise.

##### FC analysis

Given the functional anatomy of DMN [18, 40], five pairs of brain areas are selected as regions of interest (ROIs): IPL, mPFC, HF+, PCC, and LTC. The masks of ROIs were generated by the WFU Pick Atlas (http://fmri.wfubmc.edu/software/PickAtlas) corresponding to the anatomical regions embedded in the automated anatomical labelling (AAL). The mean time courses are extracted from each ROI by averaging the time courses over all voxels within the ROI. To better illustrate the functional organization of DMN subregions, whole-brain and ROI-wise FC will be employed. For whole-brain FC, Pearson correlation coefficients will be calculated between each ROI and the whole brain areas embedded in the AAL atlas [41]. After Fisher’s *z* transformation, an individual FC map will be used for group-wise comparison. For ROI-wise FC, Pearson correlation coefficients will be calculated between the corrected time series of the ROIs. The absolute value of the correlation coefficient (*r*) will be used as a measure to evaluate the FC between the ROIs [42–44]. Smaller value of FC indicates decreased connectivity between DMN subregions.

#### Covariates

Sociodemographic factors (age, sex, educational level, occupational attainment, employment status, marital status, and social network), physical comorbidities (as assessed by Chronic Illness Rating Scale), health behaviours (smoking and drinking, type and amount of other leisure activities, including social, physical, recreational, and intellectual activities, as assessed by Lifestyle Questionnaire [45]), and adherence to intervention (assessed based on the records of their work) will also be measured and included in the analysis.

### Participant timeline {13}

**Fig. 1:**
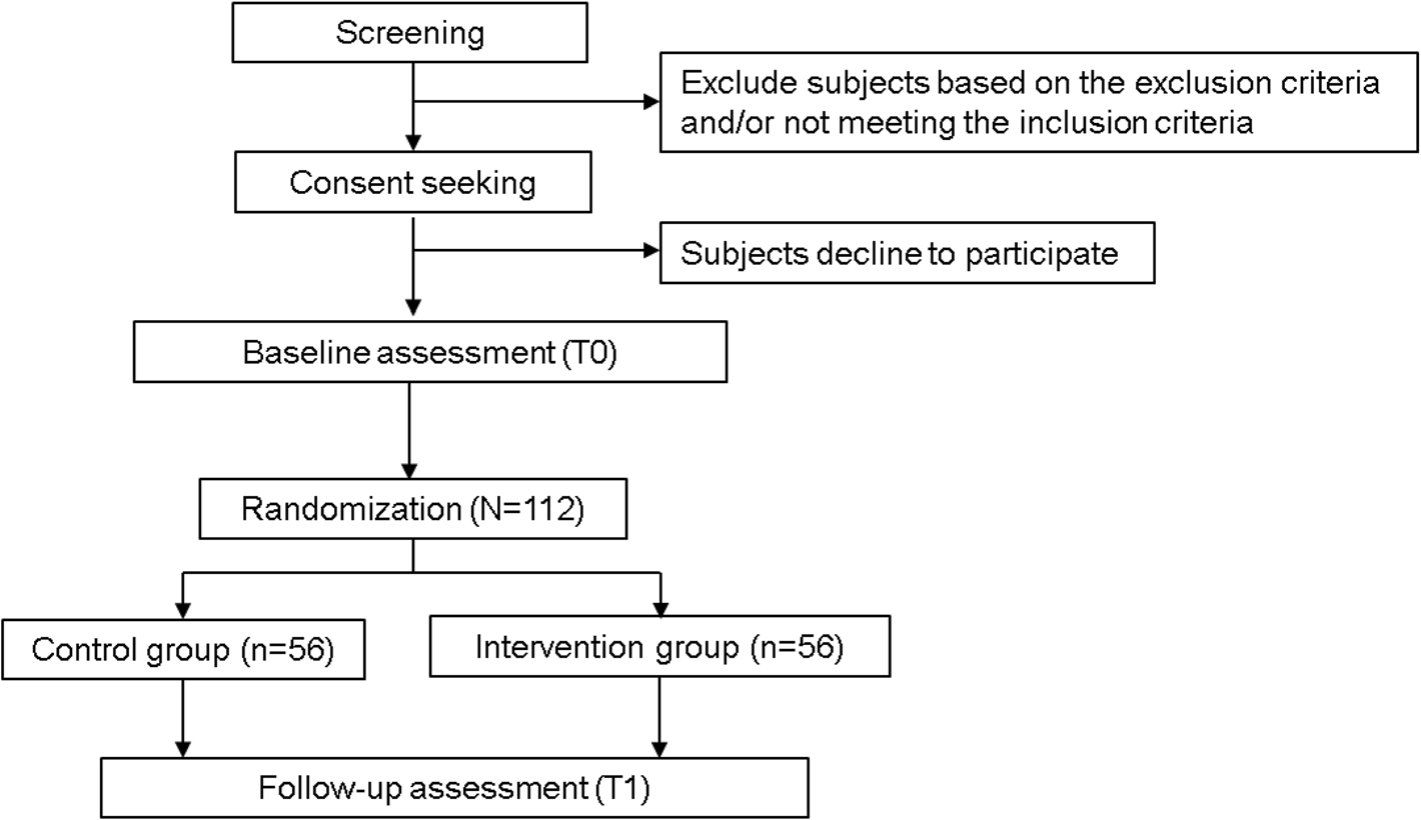
Study flowchart

The study flowchart is illustrated in Fig. 1.

### Sample size {14}

#### Cognitive function

The sample size (alpha 0.05, power 0.80) required to detect significant differences in global cognitive function over 6 months as assessed by ADAS-Cog between intervention and control groups is estimated using G*Power 3.1. Based on a similar RCT of 8-week calligraphy training in local older people with MCI living in care homes [26], the effect size (ES) on cognitive outcome was estimated to be 0.56. However, because the participants of this study are younger, less frail, living actively in the community, and at an earlier stage of AD, a relatively larger cognitive response (ES=0.70) is anticipated as suggested by a previous review of complex mental activity on cognition [46]. Nevertheless, because the control group will have ongoing calligraphy practice, the additional cognitive benefits arising from doubling the participation in calligraphy in the intervention group is anticipated to be lowered by a conservative estimate of 50%. Taking into consideration the effect of covariates on the cognitive outcome and an estimated drop-out rate of 20%, a total of 112 participants will be required for this study.

#### Functional connectivity

The sample size is calculated based on a RCT of cognitive training on memory in people with MCI (*n*=72) in which individual FC maps for the hippocampal seed were generated based on correlations between the mean signal time course within each seed region and the rest of the brain [47]. Cognitive training was found to increase FC between the hippocampus and left superior frontal lobe compared to sham, and a positive correlation (*r*=0.33; *p*=0.005) between changes in hippocampal FC and changes in the memory domain was identified. Therefore, we estimate that a sample of 70 will achieve a power of 0.80 with alpha 0.05 (two-tailed). Assuming the same dropout rate, our initial calculation of 112 participants will yield enough power for the FC-cognition analysis.

### Recruitment {15}

Participants will be recruited through our long-time collaborating network of elderly social centres in Hong Kong. Centre staff will help identify eligible individuals and refer to us for screening. The recruitment period is between January 2020 and December 2021.

## Assignment of interventions: allocation

### Sequence generation {16a}

In the randomization procedure, each participant will be assigned a special code generated by a computer (www.randomization.com). The statistician of the department will perform all the randomization.

### Concealment mechanism {16b}

Randomization will be occurred using a computer randomisation programme, so there is no human involvement, and the process will be fully concealed from both study investigators and prospective participants until the study arm is assigned.

### Implementation {16c}

The research assistants will enrol participants. The statistician of the department will generate the allocation sequence, and another independent research staff will assign participants to interventions.

## Assignment of interventions: Blinding

### Who will be blinded {17a}

Participants are assumed to be not blinded to the intervention. Nevertheless, they will be instructed not to discuss their allocation with other participants or outcome assessors. To reduce the possible expectancy bias, participants will be explained at the time of giving consent that there is no evidence to support a difference in cognitive benefit between the two arms. The research assistant who assists in consent seeking and monitoring of progress and adverse events will not be involved in outcome assessment. The assessor who will be another trained research assistant will be blinded to the randomization status and will not be involved in the intervention. The statistician responsible for the randomization is not involved in other parts of the study including data analysis.

### Procedure for unblinding if needed {17b}

In rare circumstances when the intervention has to be stopped immediately because the participant experiences an adverse event that is assessed to be related to the intervention, the principal investigator will unblind and reveal the participant’s allocation status. Unblinding will be reported to the Joint Clinical Research Ethics Committee of the Chinese University of Hong Kong and the New Territories East Cluster of the Hospital Authority.

## Data collection and management

### Plans for assessment and collection of outcomes {18a}

Screening of eligible participants, consent seeking, and cognitive outcome assessments will be performed by trained research assistants in our research centre, whereas the MRI brain scan will be performed in the teaching hospital.

A screening checklist will be used to ensure that the eligible individuals fulfil all the inclusion criteria and do not meet the exclusion criteria. Also, the number of years and the average number of hours/week of calligraphy practice that our participants have prior to joining this study will be recorded.

The cognitive outcomes and the resting-state functional connectivity, together with the covariables, will be assessed before (T0) and after the intervention (T1). Adverse events will be assessed at T1. The assessment schedule is shown in Table 1.

**Table 1:** Assessment schedule

Assessment	Baseline	Follow-up
Alzheimer’s Disease Assessment Scale-Cognitive Subscale	✓	✓
Montreal Cognitive Assessment	✓	✓
Memory Inventory for Chinese	✓	✓
Digit Span Forward	✓	✓
Digit Span Backward	✓	✓
Trail making test—Part A	✓	✓
Trail making test—Part B	✓	✓
10-min delayed recall test	✓	✓
Chinese verbal fluency test	✓	✓
Sociodemographic factors	✓	✓
Chronic Illness Rating Scale	✓	✓
Lifestyle Questionnaire	✓	✓
Neuroimaging	✓	✓
Adverse events		✓

To ensure the quality of our data (e.g. to identify any double data entry and any outliers), both the trained research assistants and the principal investigator will independently perform data cleansing before data analysis begins.

### Plans to promote participant retention and complete follow-up {18b}

To promote adherence of calligraphy practice during the 6-month intervention period, participants from both groups will be asked to use WhatsApp or email the research assistant a snapshot of their work and the amount of time they spend in calligraphy each time they finish practising. Upon receiving their work, the research assistant will acknowledge receipt of their work and log but will not give them any feedback on their work. Those who do not have smartphones or internet access will be asked to record in a diary the date and amount of time spent on their work each time they finish practising calligraphy, and to hand in their diary and work to the research assistant bi-monthly for review of adherence. Although any participant is free to withdraw from the study at any time without giving a reason, they will be asked whether they wish to be followed up according to the study schedule.

### Data management {19}

Electronic data entry will be used. The trained research assistants will collect and enter the data. Data cleansing will be performed by trained research assistants and principal investigator independently. Data will be anonymized and kept in password-protected computers. Only the principal investigator, co-investigators, and authorized research assistants will have access to the personal data during and after the study. Data management procedures are documented in the ethics application, which has already been approved by the ethics committee (Joint Chinese University of Hong Kong – New Territories East Cluster Clinical Research Ethics Committee; reference number: 2019.054).

### Confidentiality {27}

Personal identifier will not be collected in this study. The anonymized data that supports the findings of this study will be available from the principal investigator upon reasonable request. The data will be kept for 5 years after completion of study and related publications. The data will then be deleted.

### Plans for collection, laboratory evaluation and storage of biological specimens for genetic or molecular analysis in this trial/future use {33}

See above {26b} there will be no biological specimens collected.

## Statistical methods

### Statistical methods for primary and secondary outcomes {20a}

Statistical analysis will be performed using IBM SPSS Statistics for Windows. Independent *t* test (for continuous variables) and chi-square test (for categorical variables) will be used to compare socio-demographics, clinical characteristics, lifestyle activities, cognitive functions, and resting-state FCs at baseline between the control and intervention groups for exploratory analyses. Repeated measures analysis of variance will be used to evaluate the cognitive and FC changes, with time being the within-group factor, control/intervention as the between-group measure, and covariates being controlled for. Association between cognitive parameters and FCs will be computed with correlational analyses. Intention-to-treat analysis and Bonferroni correction will be adopted.

### Interim analyses {21b}

Interim analysis will be performed when approximately 10% of our sample have completed follow-up assessments. The preliminary findings will be presented in conference to promote our study.

### Methods for additional analyses (e.g. subgroup analyses) {20b}

Sensitivity analysis will be performed for participants who complete the intervention and all assessments, and a secondary analysis will be conducted to identify any differences in the effect of intervention among participants with different amounts of calligraphy practice at baseline.

### Methods in analysis to handle protocol non-adherence and any statistical methods to handle missing data {20c}

In our electronic data entry, the record cannot be submitted unless all the data have been entered, thus minimizing missing data. Adherence to intervention, which is recorded by our trained research assistants on a weekly basis, serves as a covariate and will be included in the data analysis.

### Plans to give access to the full protocol, participant-level data and statistical code {31c}

The full protocol has already been submitted to the Clinical Trials Registry (ChiCTR). The data of this study will be available from the principal investigator upon reasonable request.

## Oversight and monitoring

### Composition of the coordinating centre and trial steering committee {5d}

Weekly meetings will be held among the principal investigator and the research staff to monitor the project progress, identify and address any problems in subject recruitment, intervention delivery, and data collection, field work logistics, budget and response to major enquiries, and possible adverse events.

Monthly meetings will be held together with the co-investigators and the director of our collaborating centres to discuss the project progress, address any issues with the study logistics, review the results, and prepare for reports and publications.

To ensure good governance, quarterly meetings will be held together with the Department Chair to review project progress, outcomes, and important decisions to be made in relation to the funding and logistics, such that the project is implemented according to plan with the proposed goals achieved.

### Composition of the data monitoring committee, its role and reporting structure {21a}

The principal investigator and co-investigators will monitor the data collection and storage to ensure that the data is kept and used in accordance to the protocol. Data monitoring committee is not considered as practising calligraphy is a low-risk intervention, and our study is not supported by any sponsor.

### Adverse event reporting and harms {22}

Adverse events are expected to be minimal in this study, as no drug treatment or invasive procedure is involved, and the intervention (calligraphy) is already familiarized to the participants. Nevertheless, mild discomforts such as fatigue or muscle soreness due to prolonged sitting and writing and headache or eye soreness due to intense concentration in calligraphy practice may be experienced by some participants. These potential discomforts will be explained to participants before signing consent. Participants will be advised to report to our research assistant any health problems or suspected adverse events during the study period. All complaints with regard to our intervention will be documented, and any adverse events potentially due to our intervention will be assessed and followed by the principal investigator. The intervention will be stopped immediately if the reported event is considered to be related.

### Frequency and plans for auditing trial conduct {23}

As per the university’s requirement, an independent auditor will conduct an annual review throughout the project period.

### Plans for communicating important protocol amendments to relevant parties (e.g. trial participants, ethical committees) {25}

There is no plan for modifying the protocol at this juncture. Any proposed changes to the protocol will be subjected to review and approval by the ethics committee before adoption. Any deviations from the Protocol will be fully documented using a breach report form.

### Dissemination plans {31a}

Study findings will be published in peer-reviewed journals and disseminated to community centres. The funder has no role or restriction in the decision of publication.

## Discussion

Our anticipated findings will provide scientific evidence to the underlying mechanisms of how intellectual activities improves cognitive function. With the newer generations of older people being much better educated, being more health conscious, and having a more active and intellectually enriched lifestyle in their life course [48], their cognitive reserve is likely to be higher, and their risks of dementia are likely to be different from the previous and existing cohorts [49]. Testing whether increasing participation in habitual intellectual activities can further enhance the FC of DMN and thereby reinforce resilience to cognitive decline would be more tailored to the risk profile of this upcoming older population and can provide people with high cognitive reserve some evidence that stepping up their usual practice of intellectual activities is a useful strategy in promoting cognitive maintenance.

Due to social unrest in late 2019 and the COVID-19 pandemic since January 2020, the recruitment rate is expected to be slower than planned. Intervention is unlikely to be affected because calligraphy practice is home-based.

## Trial status

The study was initially planned to commence in September 2019 and complete in 2 years. Due to social unrest in late 2019 and the COVID-19 pandemic since January 2020, an extension of the study period has been granted, with the revised completion date on 31 December 2022.

The protocol version 1 was dated 30 December 2018, and version 2 was dated 29 February 2019. Reasons for revision are described in {3}.

Subject recruitment began on 15 January 2020 and ended on 31 December 2021.

## Abbreviations

AAL: Automated anatomical labelling
AD: Alzheimer’s disease
ADAS-Cog: Alzheimer’s Disease Assessment Scale-Cognitive Subscale
AMIC: Abbreviated Memory Inventory for Chinese
ChiCTR: Chinese Clinical Trial Registry
CVFT: Chinese verbal fluency test
DMN: Default mode network
DSB: Digit span backward
DSF: Digit span forward
ES: Effect size
FC: Functional connectivity
FOV: Field of view
HF+: Parahippocampal cortex
HK-MoCA: Hong Kong Chinese version of Montreal Cognitive Assessment
IPL: Inferior parietal lobe
LTC: Lateral temporal cortex
MCI: Mild cognitive impairment
MIC: Memory Inventory for Chinese
MNI: Montreal Neurological Institute
mPFC: Medial prefrontal cortex
PCC: Precuneus/posterior cingulate cortex
RCT: Randomized controlled trial
ROIs: Regions of interest
rs-fMRI: Resting-state functional magnetic resonance imaging
SCD: Subjective cognitive decline
sMRI: Structural MRI
SPM12: Statistical Parametric Mapping software
TE: Echo time
TMT-A: Trail making test
TR: Repetition time
WHO-ICTRP: International Clinical Trial Registration Platform of World Health Organization
